# A Type-Entry-Malperfusion-Based Propensity Score Matched Analysis Depending on Surgical Expertise in Patients Without Malperfusion Undergoing Surgery for Acute Type A Aortic Dissection

**DOI:** 10.1093/icvts/ivag020

**Published:** 2026-01-12

**Authors:** Leonard Pitts, Lina Hülsenberg, Matteo Montagner, Markus Kofler, Gaik Nersesian, Julius Kaemmel, Roland Heck, Semih Buz, Volkmar Falk, Axel Unbehaun, Jörg Kempfert

**Affiliations:** Department of Cardiothoracic and Vascular Surgery, Deutsches Herzzentrum der Charité (DHZC), Berlin 13353, Germany; Charité—Universitätsmedizin Berlin, Corporate Member of Freie Universität Berlin and Humboldt-Universität zu Berlin, Berlin 10117, Germany; DZHK (German Centre for Cardiovascular Research), Partner Site Berlin, Germany; Department of Cardiothoracic and Vascular Surgery, Deutsches Herzzentrum der Charité (DHZC), Berlin 13353, Germany; Charité—Universitätsmedizin Berlin, Corporate Member of Freie Universität Berlin and Humboldt-Universität zu Berlin, Berlin 10117, Germany; Department of Cardiothoracic and Vascular Surgery, Deutsches Herzzentrum der Charité (DHZC), Berlin 13353, Germany; Charité—Universitätsmedizin Berlin, Corporate Member of Freie Universität Berlin and Humboldt-Universität zu Berlin, Berlin 10117, Germany; Department of Cardiothoracic and Vascular Surgery, Deutsches Herzzentrum der Charité (DHZC), Berlin 13353, Germany; Charité—Universitätsmedizin Berlin, Corporate Member of Freie Universität Berlin and Humboldt-Universität zu Berlin, Berlin 10117, Germany; DZHK (German Centre for Cardiovascular Research), Partner Site Berlin, Germany; Department of Cardiothoracic and Vascular Surgery, Deutsches Herzzentrum der Charité (DHZC), Berlin 13353, Germany; Charité—Universitätsmedizin Berlin, Corporate Member of Freie Universität Berlin and Humboldt-Universität zu Berlin, Berlin 10117, Germany; Department of Cardiothoracic and Vascular Surgery, Deutsches Herzzentrum der Charité (DHZC), Berlin 13353, Germany; Charité—Universitätsmedizin Berlin, Corporate Member of Freie Universität Berlin and Humboldt-Universität zu Berlin, Berlin 10117, Germany; Department of Cardiothoracic and Vascular Surgery, Deutsches Herzzentrum der Charité (DHZC), Berlin 13353, Germany; Charité—Universitätsmedizin Berlin, Corporate Member of Freie Universität Berlin and Humboldt-Universität zu Berlin, Berlin 10117, Germany; Department of Cardiothoracic and Vascular Surgery, Deutsches Herzzentrum der Charité (DHZC), Berlin 13353, Germany; Charité—Universitätsmedizin Berlin, Corporate Member of Freie Universität Berlin and Humboldt-Universität zu Berlin, Berlin 10117, Germany; DZHK (German Centre for Cardiovascular Research), Partner Site Berlin, Germany; Department of Cardiothoracic and Vascular Surgery, Deutsches Herzzentrum der Charité (DHZC), Berlin 13353, Germany; Charité—Universitätsmedizin Berlin, Corporate Member of Freie Universität Berlin and Humboldt-Universität zu Berlin, Berlin 10117, Germany; DZHK (German Centre for Cardiovascular Research), Partner Site Berlin, Germany; Translational Cardiovascular Technologies, Institute of Translational Medicine, Department of Health Sciences and Technology, Swiss Federal Institute of Technology (ETH), Zurich 8093, Switzerland; Department of Cardiothoracic and Vascular Surgery, Deutsches Herzzentrum der Charité (DHZC), Berlin 13353, Germany; Charité—Universitätsmedizin Berlin, Corporate Member of Freie Universität Berlin and Humboldt-Universität zu Berlin, Berlin 10117, Germany; DZHK (German Centre for Cardiovascular Research), Partner Site Berlin, Germany; Department of Cardiothoracic and Vascular Surgery, Deutsches Herzzentrum der Charité (DHZC), Berlin 13353, Germany; Charité—Universitätsmedizin Berlin, Corporate Member of Freie Universität Berlin and Humboldt-Universität zu Berlin, Berlin 10117, Germany; DZHK (German Centre for Cardiovascular Research), Partner Site Berlin, Germany

**Keywords:** aortic dissection, malperfusion, aorta, surgeon, frozen elephant trunk

## Abstract

**Objectives:**

This study investigates differences in short- and mid-term outcomes in patients without malperfusion undergoing surgery for acute type A aortic dissection between specialized aortic surgeons and non-aortic surgeons.

**Methods:**

Patients who underwent surgery for acute type A aortic dissection between 2013 and 2023 defined as M0 (no malperfusion) according to the type-entry-malperfusion classification were included and divided into 2 groups according to the surgeon’s expertise: aortic surgeon vs non-aortic surgeon group, whereas an aortic surgeon was defined by expertise in extensive aortic arch surgery including frozen elephant trunk implantation on a regular basis (average ≥5/year). After propensity score matching, the groups were compared in terms of intraoperative variables and outcomes including a primary combined end-point consisting of 30-day mortality and/or CT-confirmed stroke.

**Results:**

The matched cohort comprised 2 balanced groups with 234 patients (117 in each group). Cardiopulmonary bypass, cross-clamp and distal arrest times did not differ significantly between the groups. However, more extensive aortic surgery was performed by aortic surgeons: aortic root replacement (Bentall) (*P* = .007; odds ratio [OR] 1.18 [CI, 1.05-1.32]), valve-sparing root replacement (David) (*P* = .013; OR 1.05 [CI, 1.01-1.10]), and frozen elephant trunk implantation (*P* < .001; OR 1.18 (CI, 1.09-1.27]). The combined end-point of 30-day mortality and/or CT-confirmed stroke was 26% in the non-aortic surgeon vs 23% in the aortic surgeon group (*P* = .54; OR 0.97 [CI, 0.86-1.08]). Further clinical outcomes, including 5-year survival, did not differ significantly (*P* = .170).

**Conclusions:**

Patients without preoperative malperfusion undergoing surgery for ATAAD show no differences in terms of short- and mid-term outcomes between specialized aortic and non-aortic surgeons. However, more extensive aortic repair may be performed safely by specialized aortic surgeons. These results support the definition of an aortic surgeon based on experience with the frozen elephant trunk technique and may advocate for call coverage by an aortic surgeon for type A repair at high-volume centres.

## Graphical abstract

**Figure:**
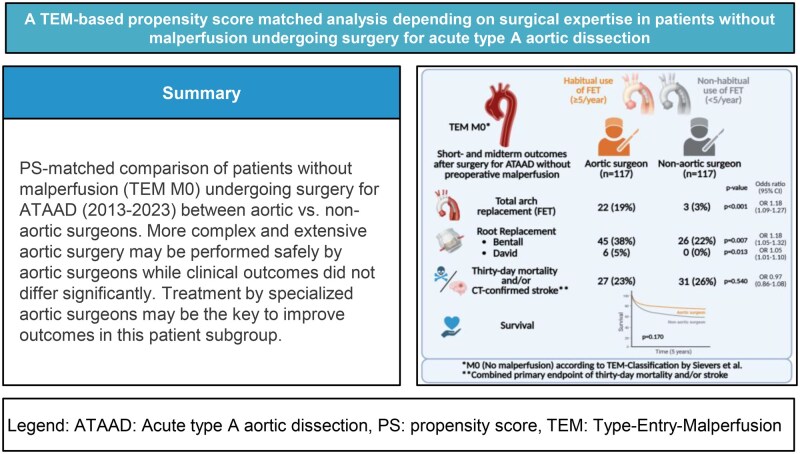

## INTRODUCTION

Acute type A aortic dissection (ATAAD) is associated with high morbidity and mortality, demanding urgent surgical repair according to current guidelines.^1^ There is solid evidence that surgical outcomes are associated with the centre-specific annual case volume of ATAAD as well as the surgeon’s individual annual case volume.^2^^,^^3^ Preoperative malperfusion in the setting of ATAAD is one of the leading risk factors for mortality and may increase the surgical complexity significantly.^4^^,^^5^ Based on this, treatment of ATAAD presenting with malperfusion should be carried out at high-volume centres—ideally by an aortic team including a specialized aortic surgeon whenever feasible—to improve outcomes.^6^^,^^7^ However, it remains a matter of debate whether this relation may also exist for patients presenting with ATAAD in the absence of malperfusion.^8^ In this propensity score matched analysis, we investigate the outcomes of patients without preoperative malperfusion undergoing surgery for ATAAD whether by a non-aortic surgeon or an aortic surgeon with corresponding expertise in extensive aortic arch surgery.

## METHODS

### Ethics approval

This study was approved by the institutional review board (No. EA2/096/20) and was conducted in accordance with the Declaration of Helsinki. The storage of participant data for multiple, indefinite research uses met the standards set forth in the WMA Declaration of Taipei. Owing to the retrospective design of the study, active informed consent was waived.

### Data availability

All relevant data are within the manuscript and its supplemental material. Further requests for data can be made to the corresponding author.

### Patient population and preoperative radiological assessment

All patients who underwent emergent surgery for ATAAD between 2013 and 2023 and had an available preoperative CT scan were included in the primary study cohort. Iatrogenic and subacute/chronic dissections (onset ≥14 days) were excluded. In addition to aortic branch vessel involvement, CT scans were analysed in terms of classification for the dissection type (T), corresponding entry site (E), and radiological ± clinical signs of malperfusion (M±) following the recently published type-entry-malperfusion (TEM) classification.^9^ Based on this, patients presenting with coronary (M1+/−), supra-aortic (M2+/−) and spinal/visceral/renal/iliac (M3+/−) malperfusion were excluded, resulting in patients exclusively classified as M0 (no malperfusion) representing the final study cohort. The definition of aortic arch anomalies included bovine aortic arch, isolated left vertebral artery or lusoric artery.

### Aortic and non-aortic surgeon

Instead of defining an aortic surgeon by a threshold annual case volume of ATAAD, we defined them by their corresponding expertise in extensive aortic arch surgery including the capability to perform frozen elephant trunk (FET) procedures on a regular basis. Surgeons who regularly performed FET implantations (average ≥5/year) electively and in the setting of ATAAD during the study period were categorized as aortic surgeons. Based on this, patients were either assigned to the aortic surgeon group (*n* = 4) or to the non-aortic surgeon group (*n* = 23).

### Surgical procedure

Following median sternotomy, full heparinization and initiation of cardiopulmonary bypass, systemic cooling was begun. Hypothermia was generally maintained at the level of moderate hypothermia (28°C) and combined with antegrade selective cerebral perfusion (either unilateral or bilateral) during caudal circulatory arrest. Retrograde cerebral perfusion was used only in a small number of cases and exclusively under deep hypothermia. A clamped anastomosis was restricted to selected cases with DeBakey type 2 dissection not extending into the aortic arch. The main arterial cannulation site was the right axillary artery. After induction of cardioplegic arrest and inspection of the entry site, the ascending aorta was resected until zone 0 or zone 1 for hemiarch replacement with an open distal anastomosis. Based on the preoperative CT scan and according to the surgeon’s decision, additional Ascyrus Medical Dissection Stent (AMDS; Artivion, Atlanta, GA, USA) implantation was performed since 2018 in selected patients.^10^ Total arch replacement with the FET technique was performed in case of an aneurysm or a located entry tear in the arch or descending aorta which was not amenable to more conservative resection. The proximal anastomosis was made in accordance with aortic root surgery: when feasible, the native root was preserved and reconstructed using felt-reinforced sutures and glue in a sandwich technique. If the aortic valve was deemed repairable, it was conserved through commissural resuspension. Valve-sparing root replacements were restricted to isolated cases and primarily performed in young patients. In all remaining situations, a composite aortic valve and root replacement with a valved conduit and coronary reimplantation was performed. Concomitant coronary artery bypass grafting was restricted to selected patients as a bailout technique in case of low cardiac output at the end of the operation.^11^

### Outcomes

The primary end-point was defined as a combined end-point consisting of thirty-day mortality and/or CT-confirmed stroke (ischaemic and/or haemorrhagic stroke and/or cerebral oedema). Secondary end-points included revision for bleeding or for malperfusion and overall survival. Revision for bleeding was undertaken in cases of pericardial tamponade and/or haemothorax. Revision for malperfusion encompassed stenting of the aorta or affected branch vessels, surgical intervention on aortic branch vessels and any form of visceral surgery required to address postoperative visceral malperfusion. The follow-up included postoperative appointments in our aortic department and telephone calls. This included survival and aortic-related reinterventions for the matched cohorts. Median follow-up was 1028 days and was closed in June 2025. Follow-up did not differ between treatment groups (1007 vs 1044 days, *P* = .63).

### Statistical analysis

**Figure 1.**
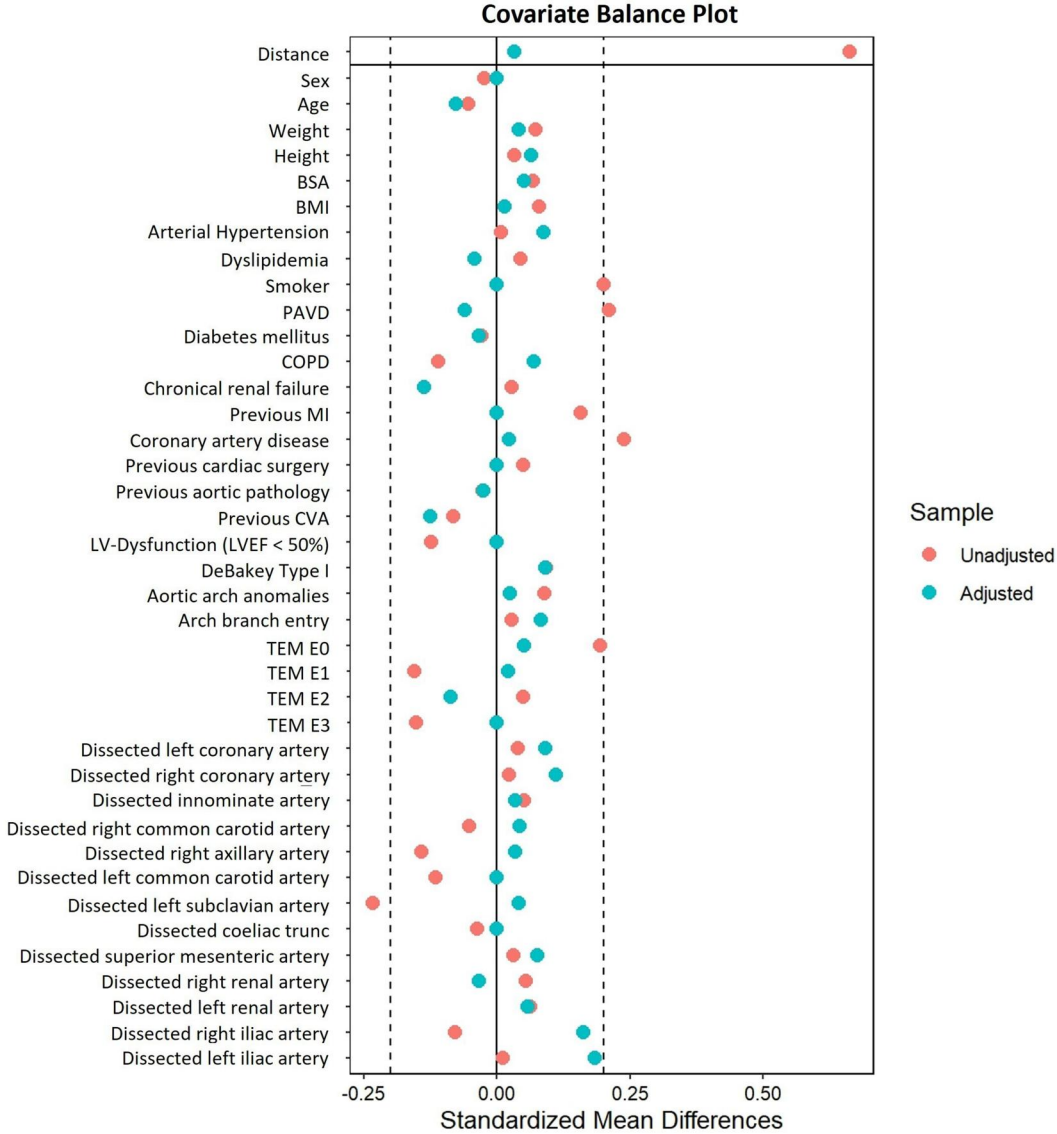
Covariate Balance Plot. Abbreviations: BMI = body mass index, BSA = body surface area, COPD = chronic obstructive pulmonary disease, CVA = cerebrovascular accident, LVEF = left ventricular ejection fraction, MI = myocardial infarction, PAVD = peripheral arterial vascular disease, TEM, type-entry-malperfusion

Shapiro-Wilk tests and histograms were used for testing the normal distribution of all continuous variables, which were not normally distributed, and therefore reported as medians with interquartile ranges (25th-75th percentiles) and compared using the Wilcoxon rank-sum test (paired Wilcoxon test for matched data). Categorical variables were presented as absolute counts and percentages, and comparisons were performed using the chi-squared test or Fisher’s exact test when expected frequencies were low (McNemar test for matched data). The MatchIt-Package version 4.5.5 from R (The R Foundation for Statistical Computing) was used for propensity score matching (1:1, “nearest neighbour” technique, caliper = 0.2), considering all preoperative variables listed in **Table 1** and **Table 2** to balance the groups regarding important preoperative variables as well as potential confounders. Intraoperative variables were not included in the matching process since they were directly associated with the surgeon’s experience. The standardized mean difference was defined as acceptable until ±0.20 and is shown in **Figure 1** as a covariate balance plot. The intraoperative variables and outcomes were compared using logistic regression analysis, generating the odds ratio (OR) with 95% CI. No adjustment for multiplicity was performed. Survival was illustrated using Kaplan-Meier curves over a period of 5 years including log-rank tests to compare survival. The α-level was defined at 0.05, and all *P*-values are 2 sided. There was no missing data in the dataset used for the analysis. Statistical analysis was performed using R version 4.3.2.

**Table 1.** Preoperative Variables (Post-Match)

Preoperative variables *N* (%)/median (IQR)	Total (*n* **=** 234)	Non-aortic surgeon (*n* **=** 117)	Aortic surgeon (*n* **=** 117)	*P*-value (**α = **0.05)	SMD
Sex (female)	82 (35)	41 (35)	41 (35)	.93	0.00
Age (years)	64 (54-76)	64 (54-76)	64 (53-76)	.70	−0.08
Weight (kg)	80 (72-93)	82 (70-94)	80 (73-92)	.65	0.04
Height (cm)	175 (168-180)	175 (167-180)	175 (168-180)	.65	0.06
BSA (m²)	1.98 (1.84-2.15)	2.00 (1.83-2.16)	1.98 (1.85-2.12)	.81	0.05
BMI (kg/m²)	27.2 (24.1-30.1)	26.7 (23.9-30.9)	27.4 (24.3-29.7)	.64	0.01
Arterial hypertension	190 (81)	93 (79)	97 (83)	.65	0.09
Dyslipidaemia	48 (21)	25 (21)	23 (20)	.94	−0.04
Smoker	92 (39)	46 (39)	46 (39)	.93	0.00
PAVD	10 (4)	6 (5)	4 (3)	.95	−0.06
Diabetes mellitus	17 (7)	9 (8)	8 (7)	1.00	−0.03
COPD	12 (5)	5 (4)	7 (6)	.95	0.07
Chronic renal failure	27 (12)	16 (14)	11 (9)	.78	−0.13
Coronary artery disease	31 (13)	15 (13)	16 (14)	1.00	0.02
Previous MI	16 (7)	8 (7)	8 (7)	.95	0.00
Previous CVA	13 (6)	8 (7)	5 (4)	.89	−0.12
Previous cardiac surgery	12 (5)	6 (5)	6 (5)	.95	0.00
Previous aortic pathology	29 (12)	15 (13)	14 (12)	1.00	−0.02
LV-Dysfunction (LVEF < 50%)	20 (9)	10 (9)	10 (9)	.95	0.00

Abbreviations: BMI = body mass index, BSA = body surface area, COPD = chronic obstructive pulmonary disease, CVA = cerebrovascular accident, LVEF = left ventricular ejection fraction, MI = myocardial infarction, PAVD = peripheral arterial vascular disease, SMD = standardized mean difference.

**Table 2.** Preoperative CT-Based Variables (Post-Match)

Preoperative variables *N* (%)/median (IQR)	Total (*n* **=** 234)	Non-aortic surgeon (*n* **=** 117)	Aortic surgeon (*n* **=** 117)	*P*-value (α **=** 0.05)	SMD
De Bakey type I	149 (64)	72 (62)	77 (66)	.66	0.09
Entry site					
• E0 (nondetectable)	24 (10)	11 (9)	13 (11)	.95	0.05
• E1 (ascending aorta)	183 (78)	91 (78)	92 (79)	1.00	0.02
• E2 (aortic arch)	25 (11)	14 (12)	11 (9)	.89	−0.08
• E3 (descending aorta)	2 (1)	1 (1)	1 (1)	.95	0.00
Malperfusion					
• M0 (no malperfusion)	234 (100)	117 (100)	117 (100)	1.00	0.00
Aortic arch anomalies	33 (14)	16 (14)	17 (15)	1.00	0.02
Arch branch entry	23 (10)	10 (9)	13 (11)	.89	0.08
Aortic vessel involvement					
• Right coronary artery	41 (18)	18 (15)	23 (20)	.77	0.11
• Left coronary artery	19 (8)	8 (7)	11 (9)	.89	0.09
• Innominate artery	102 (44)	50 (43)	52 (44)	.93	0.03
• Right common carotid artery	46 (20)	22 (19)	24 (21)	.94	0.04
• Right axillary artery	15 (6)	7 (6)	8 (7)	1.00	0.03
• Left common carotid artery	54 (23)	27 (23)	27 (23)	.94	0.00
• Left subclavian artery	52 (22)	25 (21)	27 (23)	.94	0.04
• Coeliac trunc	26 (11)	13 (11)	13 (11)	.95	0.00
• Superior mesenteric artery	21 (9)	9 (8)	12 (10)	.89	0.07
• Right renal artery	11 (5)	6 (5)	5 (4)	1.00	−0.03
• Left renal artery	18 (8)	8 (7)	10 (9)	.95	0.05
• Right common iliac artery	35 (15)	14 (12)	21 (18)	.67	0.16
• Left common iliac artery	47 (20)	19 (16)	28 (24)	.56	0.18

Abbreviation: SMD = standardized mean difference.

## RESULTS

### Preoperative variables

**Figure 2.**
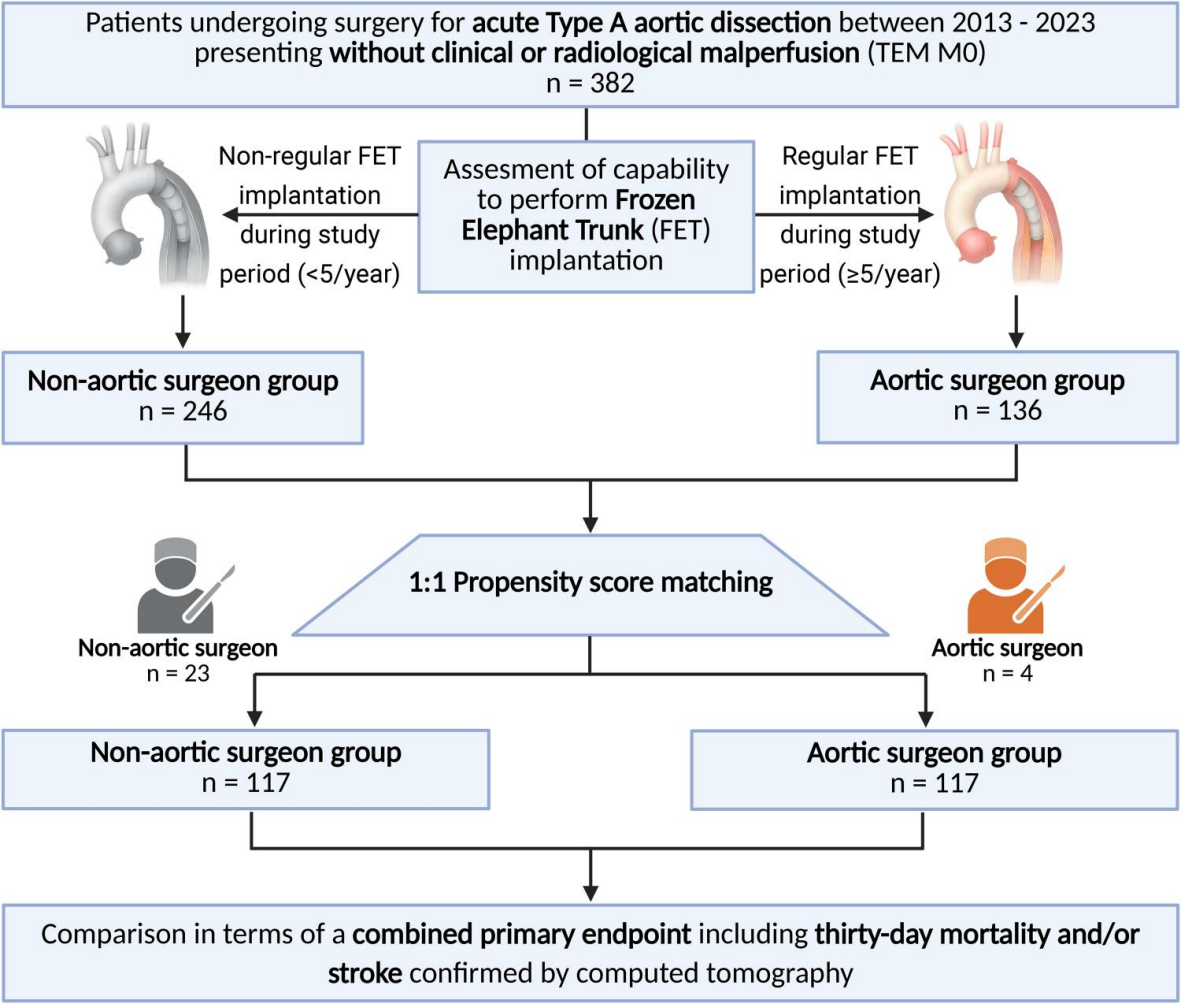
Study Flowchart. Abbreviations: FET = frozen elephant trunk; TEM, type-entry-malperfusion

After exclusion of patients presenting with any type of malperfusion according to the TEM classification, the study cohort included 382 patients: 246 patients were assigned to the non-aortic surgeon group and 136 patients to the aortic surgeon group. After 1:1 matching, the final study cohort comprised 234 patients (117 matched pairs). A corresponding study flowchart is shown in **Figure 2**. The preoperative variables for the matched cohorts are shown in **Table 1** and represent well-balanced cohorts. The preoperative CT-based variables for the matched cohorts are shown in **Table 2** and demonstrate well-balanced results between both treatment groups in terms of TEM classification including DeBakey type I dissection, the corresponding entry site and aortic branch vessel involvement from the coronary until the iliac arteries. In most patients, the corresponding entry site was in the ascending aorta/aortic root (for unmatched data, see **Tables S1** and **S2**).

### Intraoperative variables

Intraoperative variables for the matched cohorts are shown in **Table 3** (for unmatched data, see **Table S3**). Aortic surgeons primarily performed axillary arterial cannulation (*P* < .001; OR 1.12 [CI, 1.09-1.31]) and less femoral cannulation compared to non-aortic surgeons (*P* < .001; OR 0.87 [0.81-0.94]). This also accounted for bilateral antegrade cerebral perfusion (*P* < .001; OR 1.36 [CI, 1.21-1.53]). Cardiopulmonary bypass times, cross-clamp times, and distal arrest times did not differ significantly between the groups. However, more extensive surgery was performed in the aortic surgeon group: aortic root replacement (Bentall) (*P* = .007; OR 1.18 [CI, 1.05-1.32]), valve-sparing root replacement (David) (*P* = .013; OR 1.05 [CI, 1.01-1.10]) and FET implantation was performed more often compared to the non-aortic surgeon group (*P* < .001; OR 1.18 [CI, 1.09-1.27]).

**Table 3.** Intraoperative Variables (Post-Match)

Intraoperative variables *N* (%)/median (IQR)	Total (*n* **=** 234)	Non-aortic surgeon (*n* **=** 117)	Aortic surgeon (*n* **=** 117)	*P*-value (**α** **=** 0.05)	Odds ratio (95% CI)
Cardiopulmonary bypass time (min)	201 (155-262)	199 (164-262)	210 (150-266)	.86	6.22 (0.01-100)^a^
Cross-clamp time (min)	99 (78-126)	94 (79-125)	100 (77-126)	.92	0.57 (0.01-100)^a^
Circulatory arrest time (min)	35 (21-48)	35 (25-47)	35 (19-49)	.74	0.38 (0.01-99)^a^
Arterial cannulation					
• Innominate artery	12 (5)	8 (7)	4 (3)	.24	0.97 (0.91-1.02)
• Right axillary artery	195 (83)	87 (74)	108 (92)	<.001	1.12 (1.09-1.31)
• Right femoral artery	22 (9)	19 (16)	3 (3)	<.001	0.87 (0.81-0.94)
• Central	5 (2)	3 (3)	2 (2)	.65	0.99 (0.96-1.03)
Core temperature (°C)	28 (26-28)	28 (26-28)	28 (27-29)	.085	2.89 (0.87-9.58)
Unilateral ACP	104 (44)	64 (55)	40 (34)	.002	0.81 (0.72-0.92)
Bilateral ACP	86 (37)	25 (21)	61 (52)	<.001	1.36 (1.21-1.53)
RCP	20 (9)	14 (12)	6 (5)	.062	0.93 (0.87-1.00)
Clamped anastomosis	24 (10)	14 (12)	10 (9)	.39	0.97 (0.89-1.04)
Aortic root reconstruction	117 (50)	63 (54)	54 (46)	.24	0.93 (0.81-1.05)
Aortic root replacement (Bentall procedure)	71 (30)	26 (22)	45 (38)	.007	1.18 (1.05-1.32)
Valve-sparing root replacement (David procedure)	6 (3)	0 (0)	6 (5)	.013	1.05 (1.01-1.10)
AMDS	39 (17)	22 (19)	17 (15)	.38	0.96 (0.87-1.05)
Frozen elephant trunk	25 (11)	3 (3)	22 (19)	<.001	1.18 (1.09-1.27)
Concomitant CABG	16 (7)	8 (7)	8 (7)	1.00	1.00 (0.94-1.07)

Abbreviations: ACP = antegrade cerebral perfusion, AMDS = Ascyrus Medical Dissection Stent, CABG = coronary artery bypass grafting, RCP = retrograde cerebral perfusion, SMD = standardized mean difference.

a CI out of range (0.01-100).

### Outcomes

**Figure 3.**
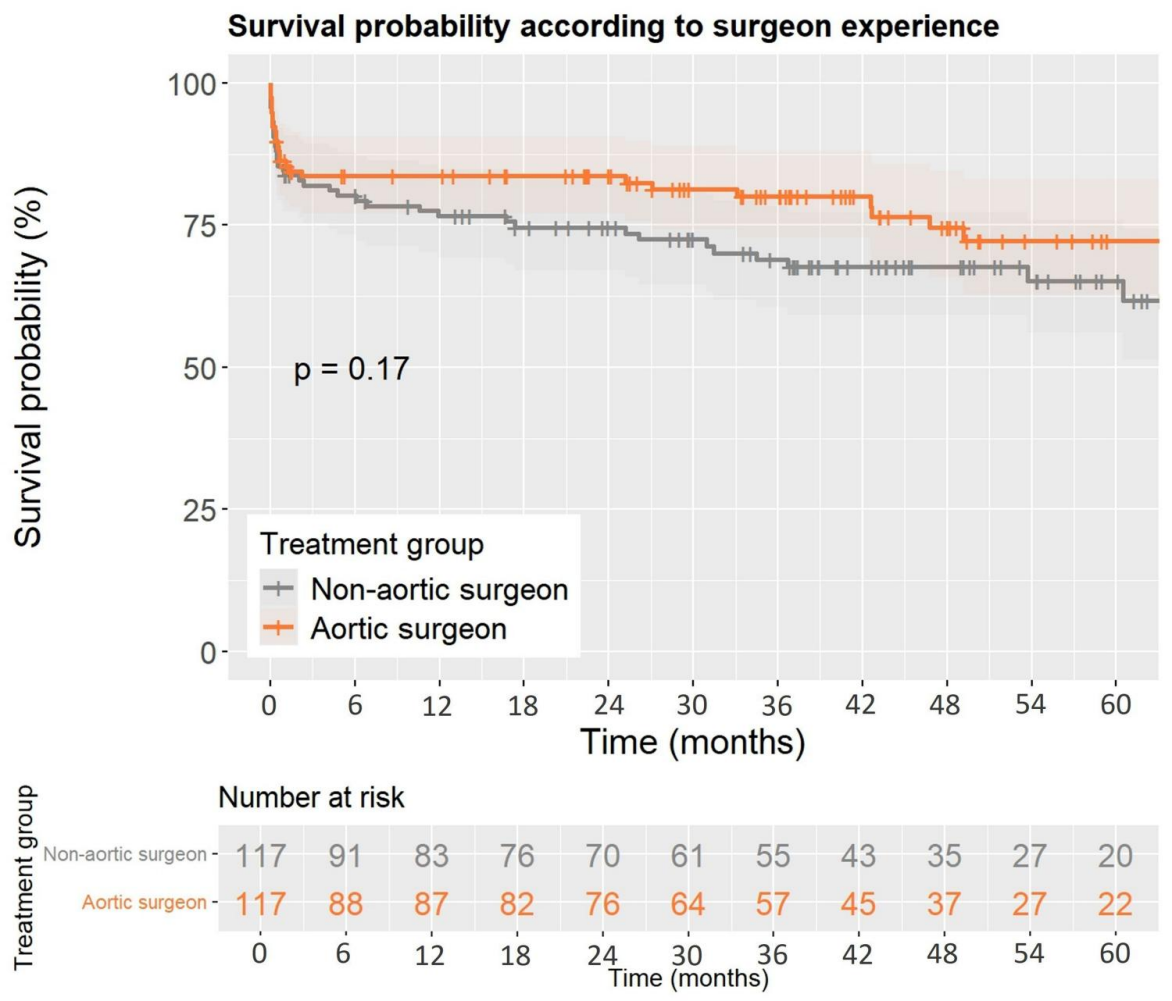
Survival according to Treatment Group

Postoperative variables for the matched cohorts are shown in **Table 4** (see unmatched cohorts in **Table S4**): the combined end-point of thirty-day mortality and/or CT-confirmed stroke accounted for 27 (23%) patients in the aortic surgeon group and 31 (26%) patients in the non-aortic surgeon group (*P* = .54; OR 0.97 [CI, 0.86-1.08)]). Incidence of aortic-related reinterventions was comparable between groups (*P* = .82; OR 1.01 [CI, 0.94-1.09]). Further secondary end-point variables and outcomes did not differ significantly. The 5-year survival is illustrated as Kaplan-Meier curve including a log-rank test for comparison in **Figure 3** and did not differ significantly between the matched cohorts (*P* = .170).

**Table 4.** Postoperative Variables (Post-Match)

Postoperative variables *N* (%)/median (IQR)	Total (*n* **=** 234)	Non-aortic surgeon (*n* **=** 117)	Aortic surgeon (n **=** 117)	*P*-value (**α** **=** 0.05)	Odds ratio (95% CI)
ICU treatment time (days)	6 (2-14)	6 (3-15)	5 (2-14)	.97	0.93 (0.02-44.05)
Ventilation time (days)	2 (1-10)	2 (1-9)	2 (1-10)	.81	0.69 (0.03-13.86)
Re-intubation	30 (13)	20 (17)	10 (9)	.051	0.92 (0.84-1.00)
Tracheotomy	38 (16)	21 (18)	17 (15)	.48	0.97 (0.88-1.06)
Open chest treatment	23 (10)	14 (12)	9 (8)	.27	0.96 (0.89-1.03)
Postoperative VA-ECLS	13 (6)	9 (8)	4 (3)	.155	0.96 (0.90-1.02)
Postoperative CRRT	26 (12)	11 (9)	15 (14)	.31	1.04 (0.96-1.14)
Revision for bleeding	47 (20)	27 (23)	20 (17)	.26	0.94 (0.85-1.04)
Revision for malperfusion	24 (10)	11 (9)	13 (11)	.67	1.02 (0.94-1.10)
Delirium	82 (35)	43 (37)	39 (33)	.59	0.97 (0.85-1.09)
Spinal ischaemia	2 (1)	0 (0)	2 (2)	.157	1.02 (0.99-1.04)
Thirty-day mortality and/or CT-confirmed stroke	58 (25)	31 (26)	27 (23)	.54	0.97 (0.86-1.08)
• Thirty-day mortality	35 (15)	19 (16)	16 (14)		
• CT-confirmed stroke	32 (14)	16 (14)	16 (14)		
Aortic-related reintervention	21 (9)	10 (9)	11 (9)	.82	1.01 (0.94-1.09)

Abbreviations: CRRT = continuous renal replacement therapy, ICU = intensive care unit, VA-ECLS = veno-arterial extracorporeal life support.

## DISCUSSION

This study revealed no differences in short- or mid-term outcomes in case of treatment by a specialized aortic surgeon vs non-aortic surgeon in patients undergoing surgery for ATAAD presenting without malperfusion. However, the results demonstrate that more radical and complex aortic surgery may be performed safely by surgeons with corresponding expertise in the FET technique, which might be an important factor to improve long-term outcomes. This underlines the relevance of providing an armamentarium of extensive aortic arch surgery to address surgical challenges which may occur in the setting of ATAAD due to its dynamic nature—also in the absence of malperfusion. While there is a general agreement that the complexity of ATAAD treatment rises in case of preoperative malperfusion demanding urgent surgical treatment by a dedicated aortic surgeon and team, it remains a matter of debate if this association may also account for patients presenting without malperfusion. These cases have been described as “stable” vs “unstable” or even “uncomplicated” vs “complicated” ATAAD in the past, which might erroneously imply less complexity and a lower operative risk for these patients in general.^8^^,^^12^ Indeed, preoperative malperfusion—especially in cases of multiple malperfusion syndromes—has been shown to be one of the driving factors for mortality in addition to advanced age in patients undergoing surgery for ATAAD.^7^^,^^13^ Great research efforts have been made in the past by investigating risk factors and different treatment concepts in patients with preoperative malperfusion.^14^^,^^15^ Based on these results, the treatment algorithm may differ in selected patient subgroups for treatment of specific malperfusion syndromes (eg, visceral malperfusion) prior to central repair and is also reflected in the current guidelines.^1^ It seems inevitable that treatment of these patients should exclusively be performed in high-volume centres by specialized aortic teams led by an aortic surgeon with corresponding expertise and surgical routine. Though patients without malperfusion undergoing surgery for ATAAD show more favourable outcomes *per se* due to the fact of less preoperative organ dysfunction, it remains unclear how postoperative outcomes might be improved in this specific subgroup.

There is solid evidence that mortality and stroke are strongly associated with the centre-specific annual case volume for ATAAD as well as the surgeon’s individual annual case volume.^3^ In a recent meta-analysis including 140 studies, the authors calculated an optimal annual case volume of at least 38 ATAAD cases per year, whereas the number needed to treat to save a patient’s life is 21 patients being transferred to a high-volume instead of a low-volume centre.^2^ However, outcomes in high-volume centres may also depend on the aortic surgeon and team on-call. In a single-centre risk-adjusted analysis of a high-volume centre including 580 patients, in-hospital mortality was significantly lower in patients who underwent surgery by a high-volume aortic surgeon (defined as >10 ATAAD cases per year) compared to a low-volume aortic surgeon.^6^ The authors concluded that outcomes at high-volume centres may be predominantly influenced by surgeon experience and not only from centre-specific resources. Referral of patients without malperfusion to a high-volume centre including surgical treatment by an aortic surgeon may be advantageous under these circumstances but will always carry the risk for aortic rupture or new onset malperfusion.^8^

Based on our propensity score matched results, we believe that an aortic surgeon may not solely be defined by the annual ATAAD case volume but by the capability to perform FET implantation. Besides the case volume, the ability to perform habitual FET implantation in the setting of ATAAD might be an adequate indicator for the definition of an aortic surgeon. This may include handling complex dissection patterns which are not adequately reflected by the TEM classification, and furthermore, adapting the extent of resection intraoperatively. The fact that more extensive and demanding aortic repair can be performed by experienced aortic surgeons while maintaining the same level of safety may support the potential treatment benefit for patients undergoing surgery for ATAAD in the absence of malperfusion.

### Limitations

This study is limited by its retrospective and unicentric nature, which may significantly impact the generalizability of the results. Though including a lot of important variables to adjust for confounders, it cannot be excluded that the comparison is affected by undetected bias not considered in the matching process. Additionally, standardized mean differences for adjusted variables were not all <±0.10 which would be the desired value for optimal matching quality. Unfortunately, long-term follow-up was not provided, potentially underestimating the treatment effects by specialized aortic surgeons on outcomes.

## CONCLUSIONS

Patients without preoperative malperfusion undergoing surgery for ATAAD show no differences in terms of short- and mid-term outcomes between specialized aortic and non-aortic surgeons. However, more extensive aortic repair may be performed safely by specialized aortic surgeons. These results support the definition of an aortic surgeon based on experience with the FET technique and may advocate for call coverage by an aortic surgeon and aortic team for type A repair at high-volume centres.

## Supplementary Material

ivag020_Supplementary_Data

## Data Availability

The data underlying this article are available in the article and in its online supplementary material.
