# Supplementary material for: A Type-Entry-Malperfusion-Based Propensity Score Matched Analysis Depending on Surgical Expertise in Patients Without Malperfusion Undergoing Surgery for Acute Type A Aortic Dissection
Source: Interdiscip Cardiovasc Thorac Surg. 2026 Jan 12;41(1):ivag020. doi: 10.1093/icvts/ivag020 (PMC12821359; doi:10.1093/icvts/ivag020)
Supplement: ivag020_Supplementary_Data [file ivag020_supplementary_data.zip › Supplemental material.docx]

**Supplemental material**

**Supplemental Table 1) Preoperative variables (pre-match)**

| **Preoperative variables**  N (%) / Median (IQR) | **Total**  **(n = 382)** | **Non-Aortic surgeon**  **(n = 246)** | **Aortic surgeon**  **(n = 136)** | **p-value**  **(α = 0.05)** | **SMD** |
| --- | --- | --- | --- | --- | --- |
| Sex (female) | 132 (35) | 86 (35) | 46 (34) | 0.82 | -0.02 |
| Age (years) | 64 (53 – 76) | 64 (54 – 76) | 63 (53 – 76) | 0.73 | -0.05 |
| Weight (kg) | 80 (72 – 92) | 80 (70 – 92) | 80 (73 – 92) | 0.58 | 0.07 |
| Height (cm) | 175 (168 – 180) | 175 (168 – 181) | 175 (168 – 180) | 0.83 | 0.03 |
| BSA (m²) | 1.99 (1.84 – 2.15) | 1.98 (1.83 – 2.15) | 1.99 (1.86 – 2.13) | 0.64 | 0.07 |
| BMI (kg/m²) | 26.7 (24.2 – 29.4) | 26.4 (24.1 – 29.4) | 27.3 (24.4 – 29.6) | 0.47 | 0.08 |
| Arterial hypertension | 311 (81) | 200 (81) | 111 (82) | 0.94 | 0.01 |
| Dyslipidemia | 77 (20) | 48 (20) | 29 (21) | 0.67 | 0.04 |
| Smoker | 144 (38) | 84 (34) | 60 (44) | 0.054 | 0.20 |
| PAVD | 19 (5) | 7 (3) | 12 (9) | 0.010 | 0.21 |
| Diabetes mellitus | 27 (7) | 18 (7) | 9 (7) | 0.80 | -0.03 |
| COPD | 32 (8) | 23 (9) | 9 (7) | 0.36 | -0.11 |
| Chronic renal failure | 40 (10) | 25 (10) | 15 (11) | 0.79 | 0.03 |
| Coronary artery disease | 45 (12) | 21 (9) | 24 (18) | 0.008 | 0.24 |
| Previous MI | 18 (5) | 8 (3) | 10 (7) | 0.070 | 0.16 |
| Previous CVA | 21 (5) | 15 (6) | 6 (4) | 0.49 | -0.08 |
| Previous cardiac surgery | 17 (4) | 10 (4) | 7 (5) | 0.62 | 0.05 |
| Previous aortic pathology | 50 (13) | 33 (13) | 17 (12) | 0.80 | -0.03 |
| LV-Dysfunction (LVEF < 50%) | 36 (9) | 26 (11) | 10 (7) | 0.30 | -0.12 |
| BMI = Body mass index, BSA = Body surface area, COPD = Chronic obstructive pulmonary disease, CVA = Cerebrovascular accident, LVEF = Left ventricular ejection fraction, MI = Myocardial infarction, PAVD = Peripheral arterial vascular disease, SMD = standardized mean difference | | | | | |

**Supplemental Table 2) Preoperative CT-based variables (pre-match)**

| **Preoperative variables**  N (%) / Median (IQR) | **Total**  **(n = 382)** | **Non-Aortic surgeon**  **(n = 246)** | **Aortic surgeon**  **(n = 136)** | **p-value**  **(α = 0.05)** | **SMD** |
| --- | --- | --- | --- | --- | --- |
| De Bakey Type I | 242 (63) | 152 (62) | 90 (66) | 0.34 | 0.09 |
| Entry site |  |  |  |  |  |
| - E0 (nondetectable) | 32 (8) | 15 (6) | 17 (12) | 0.031 | 0.19 |
| - E1 (ascending aorta) | 311 (81) | 206 (84) | 105 (77) | 0.117 | -0.16 |
| - E2 (aortic arch) | 33 (9) | 20 (8) | 13 (10) | 0.64 | 0.05 |
| - E3 (descending aorta) | 6 (2) | 5 (2) | 1 (1) | 0.33 | -0.15 |
| Malperfusion |  |  |  |  |  |
| - M0 (no malperfusion) | 382 (100) | 246 (100) | 136 (100) |  | 0.00 |
| Aortic arch anomalies | 51 (13) | 30 (12) | 21 (15) | 0.37 | 0.09 |
| Arch branch entry | 40 (10) | 25 (10) | 15 (11) | 0.79 | 0.03 |
| Aortic vessel involvement |  |  |  |  |  |
| - Right coronary artery | 68 (18) | 43 (17) | 25 (18) | 0.83 | 0.02 |
| - Left coronary artery | 31 (8) | 19 (8) | 12 (9) | 0.71 | 0.04 |
| - Innominate artery | 165 (43) | 104 (42) | 61 (45) | 0.63 | 0.05 |
| - Right common carotid artery | 81 (21) | 54 (22) | 27 (20) | 0.63 | -0.05 |
| - Right axillary artery | 34 (9) | 25 (10) | 9 (7) | 0.25 | -0.14 |
| - Left common carotid artery | 93 (24) | 64 (26) | 29 (21) | 0.31 | -0.11 |
| - Left subclavian artery | 108 (28) | 78 (32) | 30 (22) | 0.045 | -0.23 |
| - Coeliac trunc | 45 (12) | 30 (12) | 15 (11) | 0.74 | -0.04 |
| - Superior mesenteric artery | 38 (13) | 30 (12) | 18 (13) | 0.77 | 0.03 |
| - Right renal artery | 22 (6) | 13 (5) | 9 (7) | 0.59 | 0.05 |
| - Left renal artery | 32 (8) | 19 (8) | 13 (10) | 0.54 | 0.06 |
| - Right common iliac artery | 69 (18) | 47 (19) | 22 (16) | 0.48 | -0.08 |
| - Left common iliac artery | 86 (23) | 55 (22) | 31 (23) | 0.92 | 0.01 |
| IQR = interquartile range, SMD = Standardized mean difference | | | | | |

**Supplemental Table 3) Intraoperative variables (pre-match)**

| **Intraoperative variables**  N (%) / Median (IQR) | **Total**  **(n = 382)** | **Non-Aortic surgeon**  **(n = 246)** | **Aortic surgeon**  **(n = 136)** | **p-value**  **(α = 0.05)** | **SMD** |
| --- | --- | --- | --- | --- | --- |
| Cardiopulmonary bypass time (min) | 199 (163 – 256) | 197 (165 – 251) | 211 (156 – 265) | 0.48 | 0.10 |
| Cross-clamp time (min) | 99 (79 – 125) | 96 (78 – 125) | 104 (81 – 126) | 0.26 | 0.08 |
| Circulatory arrest time (min) | 35 (25 – 47) | 35 (26 – 45) | 35 (20 – 50) | 0.59 | -0.05 |
| Arterial cannulation |  |  |  |  |  |
| - Innominate artery | 21 (5) | 16 (7) | 5 (4) | 0.25 | -0.15 |
| - Right axillary artery | 309 (81) | 185 (75) | 124 (91) | <0.001 | 0.56 |
| - Right femoral artery | 43 (11) | 38 (15) | 5 (4) | <0.001 | -0.63 |
| - Central | 9 (2) | 7 (3) | 2 (1) | 0.40 | -0.11 |
| Core temperature (°C) | 28 (26 – 28) | 28 (25 – 28) | 28 (26 – 28) | 0.025 | 0.22 |
| Unilateral ACP | 187 (49) | 142 (58) | 45 (33) | <0.001 | -0.52 |
| Bilateral ACP | 125 (33) | 52 (21) | 73 (54) | <0.001 | 0.65 |
| RCP | 34 (9) | 26 (11) | 8 (6) | 0.124 | -0.20 |
| Clamped anastomosis | 36 (9) | 26 (11) | 10 (7) | 0.304 | -0.12 |
| Aortic root reconstruction | 192 (50) | 128 (52) | 64 (47) | 0.353 | -0.10 |
| Aortic root replacement (Bentall) | 103 (27) | 53 (22) | 50 (37) | 0.001 | 0.32 |
| Valve sparing root replacement (David) | 9 (2) | 1 (1) | 8 (6) | 0.001 | 0.23 |
| AMDS | 71 (19) | 50 (20) | 21 (15) | 0.241 | -0.14 |
| Frozen elephant trunk | 35 (9) | 8 (3) | 27 (20) | <0.001 | 0.42 |
| Concomitant CABG | 20 (5) | 11 (4) | 9 (7) | 0.369 | 0.09 |
| ACP = Antegrade cerebral perfusion, AMDS = Ascyrus Medical Dissection Stent, CABG = Coronary artery bypass grafting, IQR = interquartile range, RCP = Retrograde cerebral perfusion, SMD = Standardized mean difference | | | | | |

**Supplemental Table 4) Postoperative variables (pre-match)**

| **Postoperative variables**  N (%) / Median (IQR) | **Total**  **(n = 382)** | **Non-Aortic surgeon**  **(n = 246)** | **Aortic surgeon**  **(n = 136)** | **p-value**  **(α = 0.05)** | **Odds ratio**  **(95% CI)** |
| --- | --- | --- | --- | --- | --- |
| ICU treatment time (days) | 6 (3 – 15) | 6 (3 – 16) | 5 (3 – 13) | 0.66 | 0.49 (0.02-10.97) |
| Ventilation time (days) | 2 (1 – 9) | 2 (1 – 9) | 2 (1 – 10) | 0.67 | 0.58 (0.05-7.03) |
| Re-intubation | 52 (14) | 42 (17) | 10 (7) | 0.008 | 0.91 (0.84-0.97) |
| Tracheotomy | 65 (17) | 45 (18) | 20 (15) | 0.37 | 0.96 (0.89-1.04) |
| Open chest treatment | 37 (10) | 27 (11) | 10 (7) | 0.25 | 0.96 (0.91-1.03) |
| Postoperative ECLS | 18 (5) | 13 (5) | 5 (4) | 0.48 | 0.98 (0.94-1.03) |
| Postoperative CRRT | 40 (11) | 22 (10) | 18 (14) | 0.192 | 1.05 (0.98-1.12) |
| Revision for bleeding | 80 (21) | 57 (23) | 23 (17) | 0.151 | 0.94 (0.86-1.02) |
| Revision for malperfusion | 35 (9) | 18 (7) | 17 (12) | 0.09 | 1.05 (0.99-1.12) |
| Delirium | 142 (37) | 93 (38) | 49 (36) | 0.73 | 0.98 (0.89-1.09) |
| Spinal ischemia | 4 (1) | 2 (1) | 2 (1) | 0.55 | 1.01 (0.99-1.03) |
| Thirty-day mortality and/or CT-confirmed stroke | 90 (24) | 58 (24) | 32 (24) | 0.99 | 1.00 (0.91-1.09) |
| - Thirty-day mortality | 50 (13) | 33 (13) | 17 (12) |  |  |
| - CT-confirmed stroke | 53 (14) | 33 (13) | 20 (15) |  |  |
| CI = Confidence interval, CRRT = Continuous renal replacement therapy, CT = Computed tomography, VA-ECLS = Veno-arterial extracorporeal life support, ICU = Intensive care unit, IQR = interquartile range | | | | | |
